# Quantitative Longitudinal Imaging of Vascular Inflammation and Treatment by Ezetimibe in apoE Mice by FMT Using New Optical Imaging Biomarkers of Cathepsin Activity and **α**_*v*_ **β**_3_ Integrin

**DOI:** 10.1155/2012/189254

**Published:** 2012-10-17

**Authors:** Shu-An Lin, Manishkumar Patel, Donna Suresch, Brett Connolly, Bagna Bao, Kevin Groves, Milind Rajopadhye, Jeffrey D. Peterson, Michael Klimas, Cyrille Sur, Bohumil Bednar

**Affiliations:** ^1^Department of Imaging, Merck Research Laboratories, 770 Sumneytown Pike, WP44C-2, West Point, PA 19486, USA; ^2^Department of Applied Biology, Perkin Elmer, 549 Albany Street, Boston, MA 02451, USA

## Abstract

Inflammation as a core pathological event of atherosclerotic lesions is associated with the secretion of cathepsin proteases and the expression of *α*_*v*_ *β*_3_ integrin. We employed fluorescence molecular tomographic (FMT) noninvasive imaging of these molecular activities using cathepsin sensing (ProSense, CatB FAST) and *α*_*v*_ *β*_3_ integrin (IntegriSense) near-infrared fluorescence (NIRF) agents. A statistically significant increase in the ProSense and IntegriSense signal was observed within the chest region of apoE^−/−^ mice (*P* < 0.05) versus C57BL/6 mice starting 25 and 22 weeks on high cholesterol diet, respectively. In a treatment study using ezetimibe (7 mg/kg), there was a statistically significant reduction in the ProSense and CatB FAST chest signal of treated (*P* < 0.05) versus untreated apoE^−/−^ mice at 31 and 21 weeks on high cholesterol diet, respectively. The signal of ProSense and CatB FAST correlated with macrophage counts and was found associated with inflammatory cells by fluorescence microscopy and flow cytometry of cells dissociated from aortas. This report demonstrates that cathepsin and *α*_*v*_ *β*_3_ integrin NIRF agents can be used as molecular imaging biomarkers for longitudinal detection of atherosclerosis, and cathepsin agents can monitor anti-inflammatory effects of ezetimibe with applications in preclinical testing of therapeutics and potentially for early diagnosis of atherosclerosis in patients.

## 1. Introduction

Atherosclerosis, a progressive inflammatory disease, results in the development of plaques within the arterial walls that may contain intracellular and extracellular lipids, a variety of inflammatory cells, extracellular matrix, smooth muscle cells, fibroblasts, and calcification [1–4]. Acute coronary syndrome (ACS) and stroke are typically associated with the development of vulnerable plaques. A vulnerable plaque is characterized by an accumulation of macrophages and macrophage-derived foam cells, degradation of the extracellular matrix, and the formation of a necrotic core, neoangiogenesis, intraplaque hemorrhage, and a thin fibrous cap [3, 5–7]. Thus, the prevention of a heart attack or stroke would benefit from the early detection of the biological processes resulting in the inflammation and neoangiogenesis associated with atherosclerotic lesions. The noninvasive detection of atherosclerotic plaque has been clinically limited mostly to the measurements of arterial stenosis in selected arterial beds [8–11]. Such measurements have been shown to be poor predictors of clinical outcomes, thus stimulating further research into the biology of atherosclerotic lesions and development of new biomarkers with a better predictive value for the formation of vulnerable plaques [12–17]. 

 Inflammation of arterial walls is initially triggered by the accumulation of low-density lipoprotein (LDL) particles in the vessel wall and their subsequent oxidative and enzymatic modification followed by the activation of adhesion molecules and recruitment of T lymphocytes and monocytes [3]. Activated monocytes differentiate into macrophages, which accumulate modified LDL particles, evolving into foam cells [18, 19]. As atherosclerosis progresses, endothelial cells, macrophages, and foam cells die, releasing matrix metalloproteinases and other intracellular enzymes such as cathepsins [20–22]. The released proteases cleave the extracellular matrix destabilizing the plaque core and the fibrous cap [23, 24]. Transformation of monocytes into macrophages and eventually into foam cells is controlled by expression of extracellular receptors including *α*_*v*_ *β*_3_ integrin [19]. Another destabilizing factor of atherosclerotic lesions is angiogenesis, which is associated with *α*_*v*_ *β*_3_ integrin expression, and it is involved in cell-cell and cell-matrix adhesion [25]. The formation of new vessels originates from adventitial vasa vasorum and results in immature, fragile, and leaky vasculature, triggering intraplaque hemorrhage [26]. 

Molecular imaging biomarkers detecting metalloproteinase and cathepsin protease activity have been used by radiolabel-based [28] and optical imaging modalities [29] for the detection of inflammation in animal models of atherosclerosis and tissues from human atherosclerotic lesions [9, 30]. Cathepsin activatable, optical imaging agents based on cleavage of L-lysine sequences have been successfully applied in the characterization of atherosclerotic lesions in mouse and rabbit models of atherosclerosis and in the detection of cathepsin protease activity in human atherosclerotic plaques obtained at carotid endarterectomy [31–36]. Using these agents, fluorescence molecular tomography (FMT) and computed tomography (CT) have been applied to quantitatively evaluate the levels of inflammation in the mouse apoE^−/−^ models of atherosclerosis [37]. Furthermore, agents with *α*_*v*_ *β*_3_ integrin targeted ligand have been employed to detect inflammation as well as angiogenesis in atherosclerotic lesions of animal models using a broad spectrum of imaging modalities [38–43]. 

Here we describe the development and evaluation of new cathepsin activatable agents and compare them to the new *α*_*v*_ *β*_3_ integrin targeting agent in a longitudinal study of atherosclerotic apoE^−/−^ mice undergoing prophylactic treatment with the anti-inflammatory therapeutic ezetimibe [44, 45] using FMT.

## 2. Methods

### 2.1. Determination of IC_50_ in Cell Binding Assays

HEK293-*α*_*v*_ *β*_3_  cells were lifted with trypsin-EDTA and washed four times with serum-free MEM. Cells (25,000 cells/well) were added to microtiter wells coated with vitronectin or fibronectin and allowed to attach for 2 hours at 37°C in a humidified incubator in the absence or presence of increasing concentrations of IntegriSense or parent compound. Nonattached cells were gently washed away. Similar experiments were carried out with HEK293 cells stably transfected with *α*_*v*_ *β*_5_, *α*_IIb_ *β*_3_, or *α*_5_ *β*_1_. The attached cells were quantified by colorimetric detection of hexosaminidase enzymatic activity in a Vmax plate reader (Molecular Devices, Menlo Park, CA). The number of attached cells was quantified using a standard curve for each cell line assayed and expressed as a mean value of triplicate samples.

### 2.2. Murine Atherosclerosis Model and Diet

Ten-week old apoE^−/−^ female mice (*n* = 184 total: 20 for *in vivo*-*ex vivo *signal correlation study, 44 for longitudinal study, 120 for treatment study; Taconic Farms, Inc, Hudson, NY) fed on a 0.15% [wt/wt] cholesterol diet which included 9% of their caloric intake from fat (Research Diets, Inc, New Brunswick, NJ) were used as an animal model of atherosclerosis. In the longitudinal study, C57BL/6 mice (*n* = 14 total; Taconic Farms, Inc, Hudson, NY) fed on a normal chow diet served as controls. In the treatment study, apoE^−/−^ mice were fed the diet described above with or without cholesterol absorption inhibitor ezetimibe (7 mg/kg/d). All experiments were approved by our Institutional Animal Care and Use Committee (IACUC).

### 2.3. Correlation between *In Vivo* FMT Signal and *Ex Vivo* Fluorescence

Aged apoE^−/−^ mice (8 to 10 months on diet), which developed atherosclerotic lesions, were injected with ProSense 750 (5 nmol per animal), ProSense FAST 750 (8 nmol per animal), or IntegriSense 750 (4 nmol per animal, all from Perkin Elmer, Boston, MA) via tail vein 24 hr or 48 hr before FMT imaging, respectively. Images were acquired by FMT2500 (Perkin Elmer) at 750 nm wavelength. The resulting 3D dataset was reconstructed and the region of interest (ROI) was defined within the chest area (Figure 2) using the vendor's software (TrueQuant, Perkin Elmer), and the threshold was set to 30% of the max concentration within the ROI. After *in vivo* imaging, animals were euthanized with CO_2_, and their aortas were excised. Fluorescence reflectance images of the dissected arteries (carotid arteries with aortic arch and thoracic part of descending aorta) were then acquired with the FMT2500. 

### 2.4. FMT-CT Coregistration

To verify the *in vivo* FMT signal noninvasively, the mice were imaged with a GE Explore Locus Ultra (micro CT, GE) after FMT acquisition using a CT bed designed to hold the FMT animal cassette therefore ensuring the animal remained in the same position. The FMT dataset was exported in Dicom format and then co-registered to the CT dataset using Amira 5.2 (Visage Imaging, San Diego, CA).

### 2.5. Flow Cytometry

After probe-injected mice were euthanized, their aortas were excised and terminal blood samples were collected. The aortas were minced with fine scissors and dissociated into single cells by incubating with Liberase TH and TM (Roche Applied Science, Indianapolis, IN) at 37°C for 1.5 hours. The resulting cell suspensions were then passed through 40 *μ*m cell strainer (BD Biosciences, Bedford, MA) and washed with PBS. The blood samples were incubated in ACK buffer (Cambrex, Walkersville, MD) to remove blood cells and then washed with PBS. For multicolor flow cytometry analysis of lymphoid cells, myeloid cells (including monocytes/macrophages and neutrophils) and other cells (including stromal cells of the aortic wall), cells were incubated with a cocktail of mAbs (BD Biosciences, San Francisco, CA) against T cells (CD90-FITC),B cells (B220-FITC), NK cells (CD49b-FITC, NK1.1-FITC), and myeloid cells (CD11b-PE) at 4°C for 30 min and then analyzed on an LSRII (BD Biosciences, San Francisco, CA). Acquisition data was analyzed by the FlowJo software (Tree Star Inc., Ashland, OR).

### 2.6. Immunofluorescence Microscopy

To demonstrate co-localization of macrophages and the IntegriSense 680 and ProSense 680 probes, selected vessels were stained with a monoclonal anti-CD68 antibody. Ten micron-thick cryosections were prepared and fixed in ice-cold acetone/ethanol (3 : 1) for 10 min. and washed in PBS. Nonspecific binding was blocked by incubation with 5.0% nonimmune goat serum for 30 min followed by incubation with the rat anti-mouse CD68 antibody (5 *μ*g/mL; MCA-1957, AbD Serotec, Raleigh, NC) for 1 hr at room temperature. Sections were washed in PBS and incubated with AlexaFluor 488 goat anti-rat IgG (4 *μ*g/mL; Molecular Probes, Eugene, OR) for 1 hr and again washed in PBS. Sections were mounted using a fluorescent mount medium with or without DAPI counterstain and examined with a Nikon E-1000 microscope equipped with the appropriate band-pass filters.

### 2.7. Longitudinal Study Design

For the longitudinal study, both ApoE^−/−^ (*n* = 15 for each agent) and C57BL/6 (*n* = 9 for ProSense, *n* = 5 for IntegriSense) mice were injected with ProSense750 or IntegriSense750 and imaged with the FMT2500 as described above. The scans were conducted at the times indicated in the figures.

For the treatment study, ProSense, CatB FAST agent or ProSense FAST (8 nmol per animal, Perkin Elmer) were injected into apoE^−/−^ mice (both treated; *n* = 15 for each agent, and untreated; *n* = 15 for each agent) via tail vein 24 hr before imaging. IntegriSense (2 nmol per animal, Perkin Elmer) injected mice were images 48 hr after injection. The imaging acquisition was carried out at the time points as depicted in Figures 7 and 8.

### 2.8. Quantitative Immunohistochemical Analysis

After apoE^−/−^ mice were euthanized, their aortas were excised and fixed in 10% buffered formalin (Figure 6(a)). The aortas were then divided into six levels as indicated in Figure 6(b). All vessels were embedded into paraffin blocks, sectioned at 4 *μ*m, and placed onto adhesive-coated slides. Six slides per level were taken at ~50 *μ*m intervals. Slides were stained with hematoxylin and eosin to demonstrate general morphology and immunostained with mAbs against the F4/80 antigen (1 : 100, eBioscience, Inc., San Diego, CA). Slides were subsequently incubated with a peroxidase-conjugated anti-mouse IgG antibody and 3′3′-diaminobenzidine (DAB) chromogen to identify macrophages within the plaque regions and counterstained with hematoxylin (Figure 6(c)). The stained slides were analyzed using the Ariol image analysis platform (Genetix, San Jose, CA) for macrophage quantification. Regions of interest were drawn to include only the plaque area and appropriate size, and color classifiers were applied to specifically identify the DAB stained (brown) macrophages, which were then counted.

### 2.9. Serum Cholesterol Concentration

Terminal serum samples (*n* = 12) were collected at the end of the treatment study, and the HDL-C and total cholesterol levels were determined by following the manufacturer's protocol of HDL-Cholesterol E kit (Wako Chemicals, Richmond, VA) with and without adding precipitating reagent, respectively. 

### 2.10. Plasma Ezetimibe Concentration

Terminal plasma samples (*n* = 12) were collected at the end of the treatment study and assayed for and ezetimibe-glucuronide using Aria LC-MS/MS with a standard calibration range from 5 to 10,000 nM and a quantification limit of 5 nM. 

### 2.11. Statistics

Data are presented as mean ± SEM. For statistical analysis, we used ANOVA followed by Bonferroni *t*-test for multiple comparisons. Differences were considered significant at *P* < 0.05. The correlation between the *in vivo* and *ex vivo* signal was evaluated using linear regression analysis.

## 3. Results

### 3.1. Molecular and Imaging Characteristics of Cathepsin and *α*_*v*_ *β*_3_ Integrin Agents

**Figure 1:**
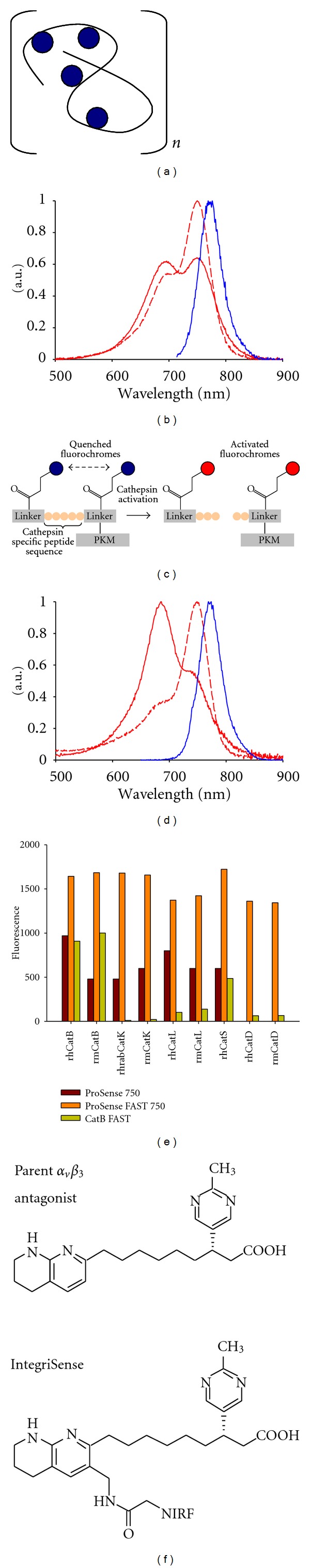
Basic characteristics of cathepsin and *α*_*v*_ *β*_3_ integrin agents. (a), (b) Cartoon and spectra of ProSense ~400 kDa polylysine-based [27]; (c), (d) Cartoon and spectra of FAST peptide-based agents ~23 kDa, PKM: pharmacokinetic modifier, absorption spectra (before activation—dash red and after activation: solid red), emission spectra: solid blue; (e), selectivity in cathepsin probe activation at 24 hours incubation; (f), Chemical structure of parent *α*_*v*_ *β*_3_ integrin ligand (top) and IntegriSense (bottom, ~1278 Da) NIRF: VivoTag fluorophore.

Structural schematics, spectral characteristics, and the activation selectivity of the cathepsin agents and a chemical structure of *α*_*v*_ *β*_3_ integrin agent used in our experiments are depicted in Figure 1. The cathepsin activated agent ProSense (Figures 1(a) and 1(b)), used in the detection of atherosclerotic lesions before [34–37], is based on ~400 kDa polylysine macromolecules with covalently attached fluorophores [27]. The newly developed cathepsin agents ProSense FAST and Cat B FAST are based on low molecular weight substrate peptides with fluorochromes attached to the C- and N-termini of the peptide resulting in 95% of the fluorescence quenching and a hypsochromic (“blue”) shift of about 60 nm in the absorption spectra (Figures 1(c) and 1(d)). The quenching of the fluorophores in the ProSense agent is achieved by tight packing of the polymer coil, which slows the activation of this agent. Thus, the full activation of the ProSense agent requires 24 hours while the newly designed peptide-based agents are fully activated within 6 to 8 hours (data no shown). The selectivity of the agents for activation by 24 hours incubation with different cathepsins is shown in Figure 1(e). Both ProSense and ProSense FAST agents were activated by most of the evaluated cathepsins, while the Cat B FAST agent was activated mostly by cathepsin B (Figure 1(e)). 

The *α*_*v*_ *β*_3_ targeted agent, IntegriSense, is based on a low molecular weight peptidomimetic ligand of *α*_*v*_ *β*_3_ integrin, which was chemically modified to covalently attach the VivoTag (NIRF) (Figure 1(f)). The affinity of the agent for activated *α*_*v*_ *β*_3_ integrin was determined to be ~ 0.5 nM [46] and the agent is highly selective for *α*_*v*_ *β*_3_ (Table 1). 

### 3.2. Agents Detected *In Vivo* Using FMT Correlate with *Ex Vivo* Fluorescence Measurements of Dissected Arteries

**Figure 2:**
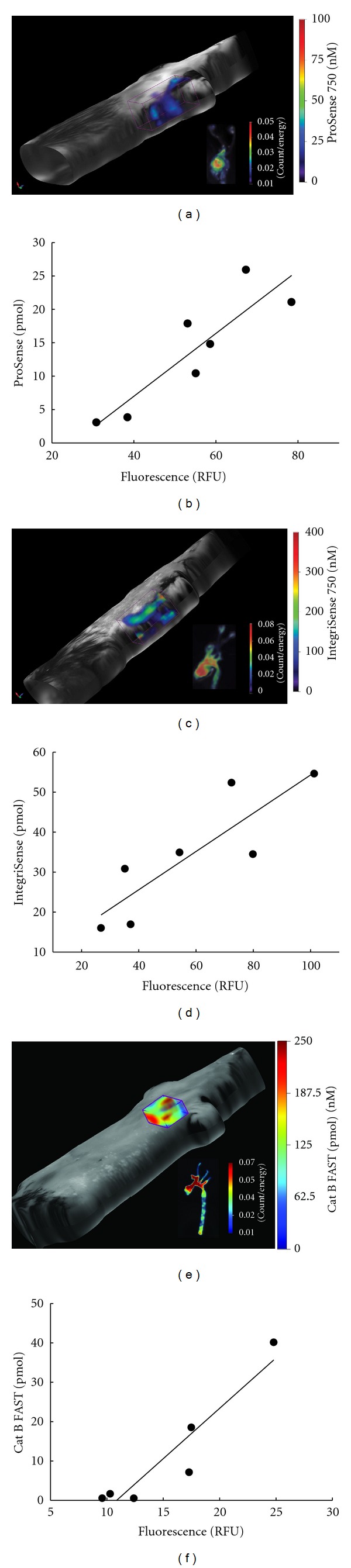
Examples of *in vivo* FMT images of apoE^−/−^ mice and *ex vivo* fluorescence images of the corresponding areas of dissected arteries and correlation between the *in vivo* and *ex vivo* measurements. (a), (b) ProSense (*r* = 0.89, *P* < 0.05); (c), (d) IntegriSense (*r* = 0.86, *P* < 0.05). (e), (f) Cat B FAST (*r* = 0.94, *P* < 0.05). Plots for ProSense and IntegriSense are based on seven and for Cat B FAST on six different animals.

To assess the severity of atherosclerotic disease, we focused on the aortic arch and the proximal portion of the descending aorta. Since there is limited anatomical information to guide *in vivo* FMT analysis, we compared fluorescent signal within the ROI drawn to capture the entire volume of the chest region with the signal measured within the ROI drawn on the excised arteries (for details see Methods) to ensure the accuracy of the *in vivo* quantification (Figures 2(a)–2(f)). The total amount of the probes (pmol) detected *in vivo* correlated well with the fluorescence (counts/energy) measured *ex vivo* for all evaluated agents (Figures 2(b), 2(d), and 2(f)). This suggests that the *in vivo* quantification reflects the actual signal from the area of interest and can therefore be used to measure the inflammation non-invasively using fluorescence of these imaging agents.

### 3.3. Coregistration of Fluorescence FMT Signal and an Anatomical CT Image

**Figure 3:**
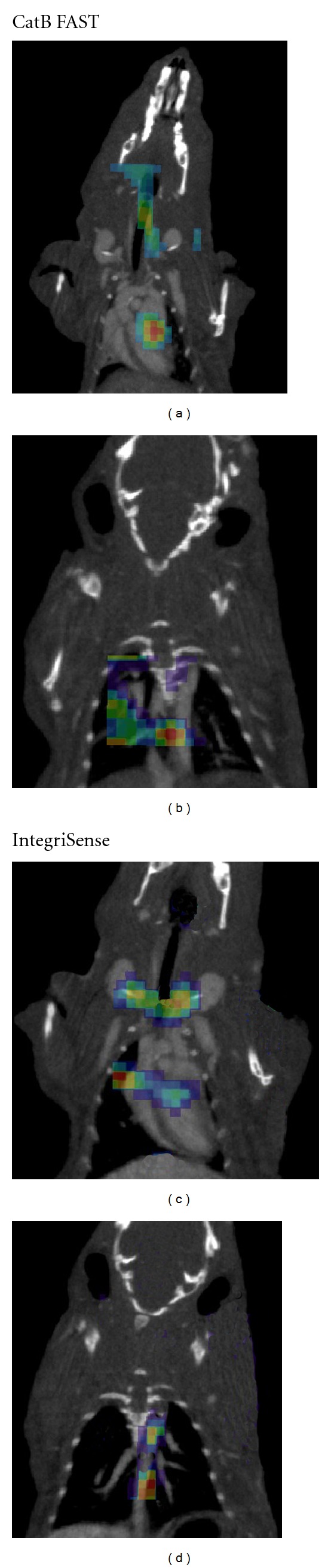
Coregistration of FMT fluorescence images with CT anatomical images. (a) (Aortic root and arch and carotid arteries), (b) (proximal part of descending aorta) using Cat B FAST, (c) (aortic root and arch and carotid arteries), (d) (proximal part of descending aorta) using IntegriSense.

To further identify the location of the signal, we co-registered the FMT signal with CT images based on fiducial landmarks that are incorporated into the animal holder and are identified by both FMT and CT. Using anatomical detail from the CT image combined with FMT molecular information, we determined that the majority of the fluorescent signal was in the aortic root, arch, and the proximal portion of the descending aorta of animals injected with cathepsin-activated (Figures 3(a) and 3(b) or *α*_*v*_ *β*_3_-targeted agents (Figures 3(c) and 3(d)).

### 3.4. Cellular Target Profile by Flow Cytometry

**Figure 4:**
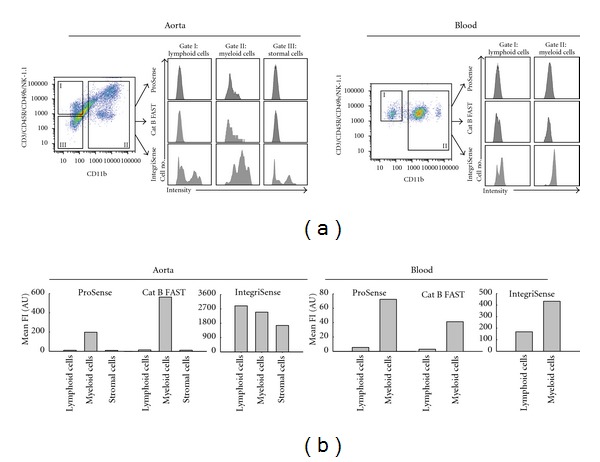
Flow cytometric analysis of the agent's fluorescence in the cells isolated from dissected arteries and blood of apoE^−/−^ mice on high cholesterol diet 24 hours after injection of cathepsin and IntegriSense agents. (a) Cells were before flow cytometry incubated with an antibody cocktail, which allows for selective identification of lymphoid, myeloid cells including monocytes/macrophages and neutrophils and other cells such as stromal cells of the aortic wall. (b) Mean fluorescence histograms of these cells show the highest distribution of cathepsin probes in the myeloid cells, while the *α*_*v*_ *β*_3_ integrin agent was found in all types of identified cells. In the blood, the relative distribution of all agents is similar. However, the absolute signal of cathepsin probes is in blood much lower than in arterial cells, while the distribution of *α*_*v*_ *β*_3_ integrin agent is higher.

To validate the cellular source of the fluorescence signal *in vivo*, we performed flow cytometry of single cell suspensions generated from digested murine aortas. Monocytes/macrophages were identified as the dominant cellular signal source for all cathepsin activated agents tested. We observed a similar labeling profile in the blood; however, the signal intensity was 10 times lower compared to the aortic signal. Fluorescence signal from the *α*_*v*_ *β*_3_ targeted agent, IntegriSense, was found not only in monocytes/macrophages but also in lymphoid and stromal cells. The signal from IntegriSense was higher in monocytes/macrophages than in lymphoid cells in the blood (Figure 4).

### 3.5. Immunofluorescence Microscopy Reveals Colocalization of Fluorescence of Optical Agents with Macrophages

**Figure 5:**
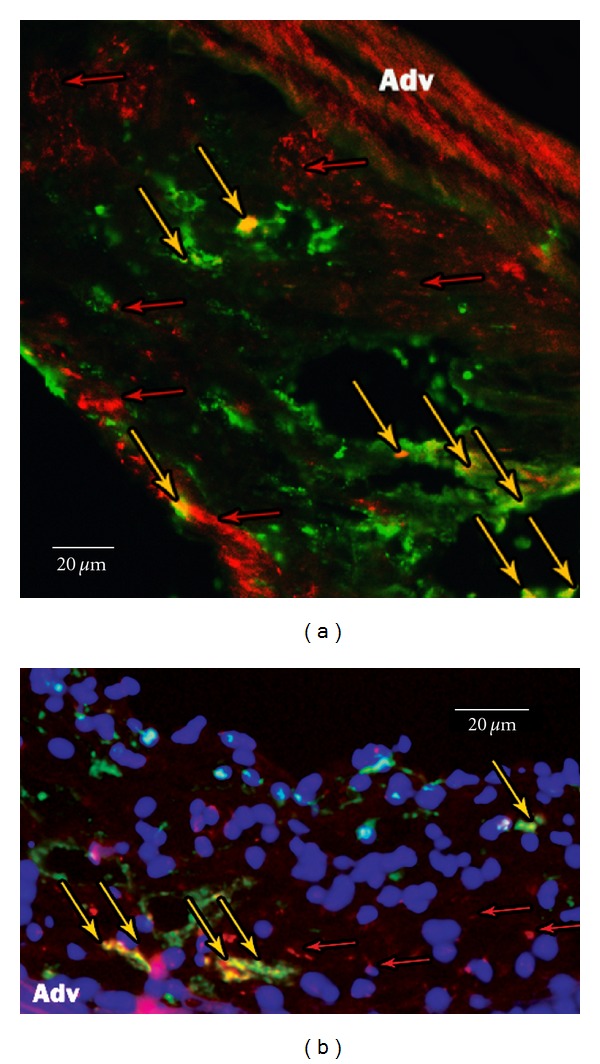
Immunofluorescence histochemistry of the sections from the aortic arch apoE^−/−^ mice on a high cholesterol diet. (a) IntegriSense, (b) ProSense, red: imaging agent, green: CD 68 antibody staining monocytes/macrophages, blue: DAPI staining of cell nucleus, yellow arrows: colocalization of agents and macrophages, red arrows: distribution of agents in the core of atherosclerotic lesion, Adv: adventitia.

The microscopic fluorescence distribution of both cathepsin activated and *α*_*v*_ *β*_3_ targeted agents was compared to immunoreactive staining for macrophages CD68 (Figure 5). There was good colocalization of fluorescence from the optical agents with immunofluorescent CD68 signal, while some of *α*_*v*_ *β*_3_ agent was found located in the adventitia.

### 3.6. *In Vivo* FMT Quantification versus *Ex Vivo* Macrophage Count

**Figure 6:**
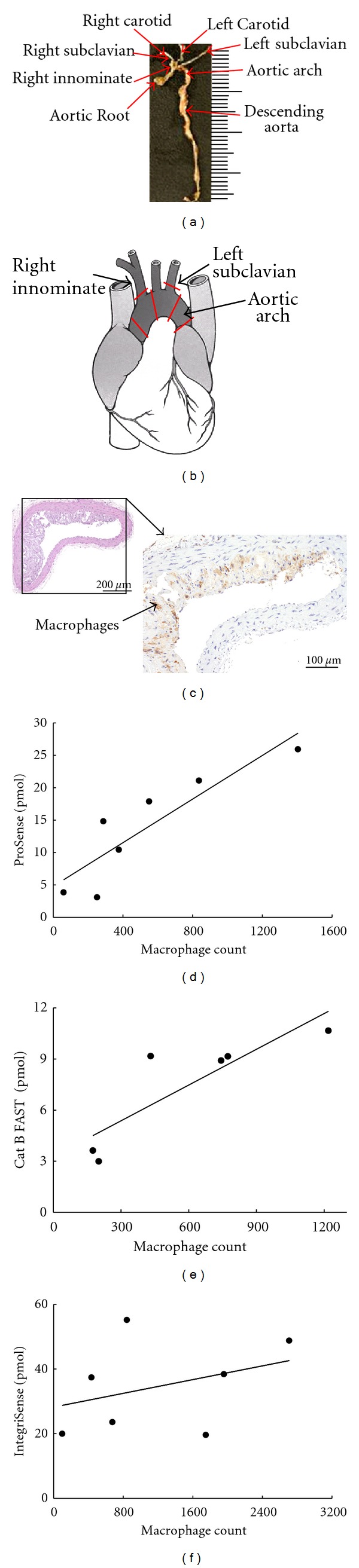
Correlation between quantitative immunohistochemical analysis of the macrophage content and the FMT measured agent amount in selected sections from apoE^−/−^ mice on high cholesterol diet. (a) An example of dissected arteries from apoE^−/−^ mice. (b) A mouse heart cartoon with six regions marked with red lines, where the arteries were sectioned for histology. (c) An example of stained section used in the macrophage quantification. (d), (e), (f) Plots of total agent amount measured *in vivo* by FMT as a function of macrophage count for ProSense ((d), *r* = 0.89, *P* < 0.05, *n* = 7), Cat B FAST ((e), *r* = 0.86, *P* < 0.05, *n* = 6), and IntegriSense ((f), *r* = 0.35, *P* = 0.44, *n* = 7), respectively.

Macrophages have been shown to play an important role in the inflammatory process of atherosclerosis [1, 2, 5, 18]. To evaluate the prognostic value of different optical agents for non-invasive detection of the disease, quantitative immunohistochemical analysis of macrophages in aortic arteries using F4/80 monoclonal antibodies was performed, and the macrophage counts were then correlated with *in vivo* FMT measurements of the same animals. Quantification of the *in vivo* FMT signal and *ex vivo* macrophage count showed a good correlation for both ProSense (*r* = 0.89, *P* < 0.05) and Cat B FAST (*r* = .86, *P* < 0.05), but not for IntegriSense (*r* = 0.35, *P* = 0.44) (Figures 6(d)–6(f)).

### 3.7. Longitudinal Imaging Using Cathepsin B Sensing and *α*_*v*_ *β*_3_ Integrin Optical Agents in apoE^−/−^ Mice

**Figure 7:**
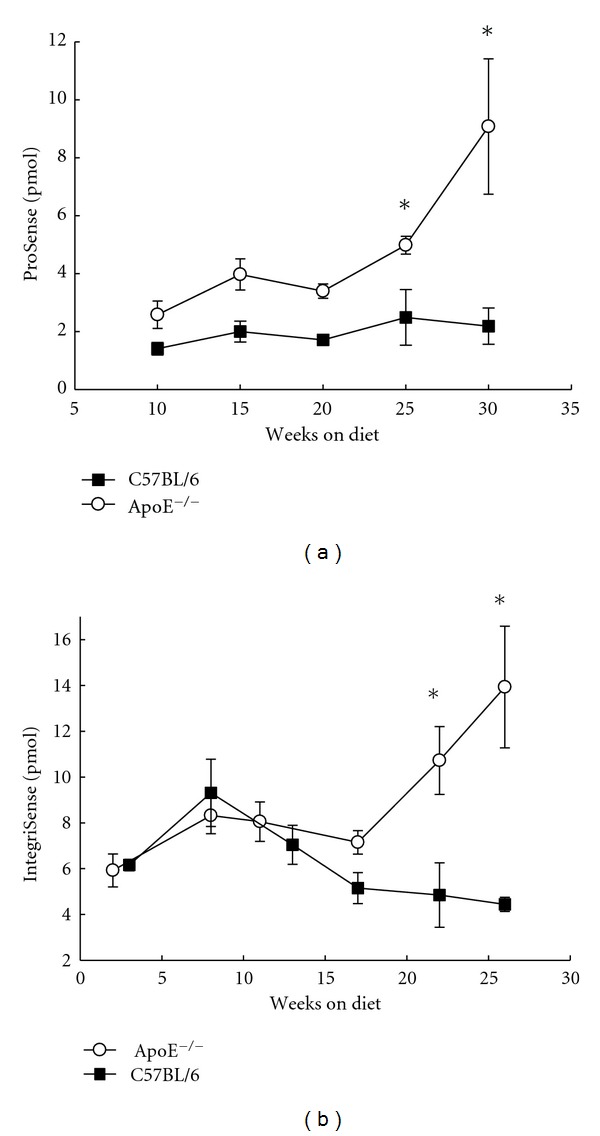
Quantitative *in vivo* FMT measurements of agents in the thorax area of apoE^−/−^ mice (*n* = 15 in each group, total 30 animals) on a high cholesterol and control C57BL/6 mice (*n* = 9 for ProSense, *n* = 5 for IntegriSense study) on regular chow diet. (a) ProSense; (b) IntegriSense (mean ± SEM, *P < 0.05).

To access cathepsin activity or *α*_*v*_ *β*_3_ integrin expression as a function of time throughout disease progression, we performed *in vivo* FMT imaging in both apoE^−/−^ and C57BL/6 mice at time points indicated in Figure 7. A statistically significant increase in the amount of ProSense and IntegriSense was observed in the chest area of apoE^−/−^ versus C57BL/6 mice starting 25 and 22 weeks on diet, respectively. 

### 3.8. Cathepsin Agents but Not *α*_*v*_ *β*_3_ Integrin Agent Detect Inhibition of Inflammation by Ezetimibe

**Figure 8:**
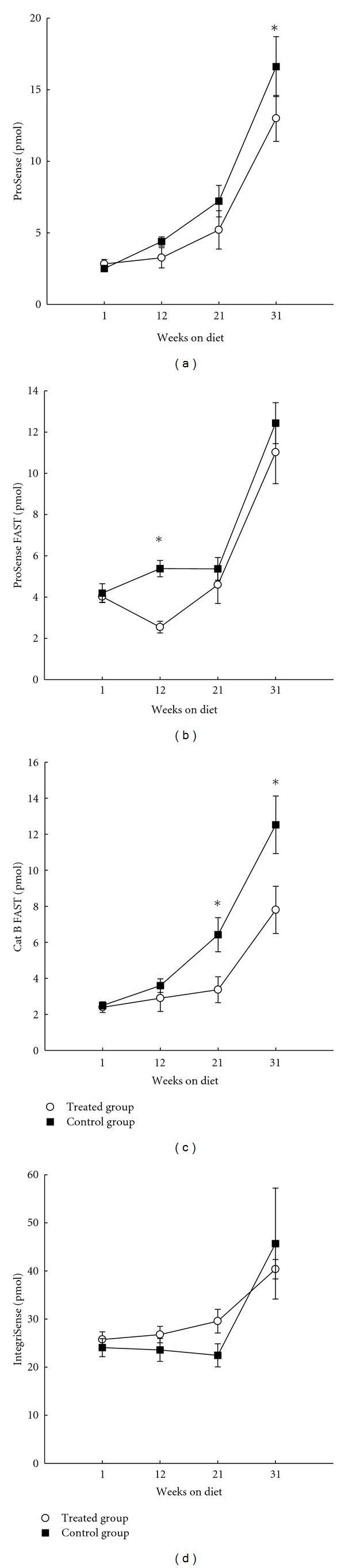
The effect of prophylactic treatment using ezetimibe on the FMT measured levels of agents in thorax areas of apoE^−/−^ mice (*n* = 15 in each group, total 120) on high cholesterol diet. (a) ProSense; (b) ProSense FAST; (c) Cat B FAST; (d) IntegriSense (mean ± SEM, *P < 0.05).

We further evaluated the newly developed agents for the detection of reduced inflammation associated with atherosclerotic lesions in apoE^−/−^ mice upon prophylactic treatment with the cholesterol lowering agent, ezetimibe. Ezetimibe is known to lower LDL and inflammation associated with the atherosclerosis in apoE^−/−^ mice [44]. In our experiments, ezetimibe lowered the LDL and elevated HDL levels about threefold (Table 2). Cat B FAST detected a statistically significant effect of the treatment at both 21 and 31 weeks on the diet (Figure 8(c)), while the large, nonselective, polymer-based agent, ProSense, identified a statistically selective effect of the treatment only at 31 weeks on diet (Figure 8(a)). The newly developed nonselective agent, ProSense FAST, found a statistically significant effect of the treatment only at 12 weeks (Figure 8(b)) while IntegriSense was insensitive to the effect of treatment by ezetimibe (Figure 8(d)). 

## 4. Discussion

Heart attack and stroke, often fatal clinical outcomes of atherosclerosis, are frequently associated with vulnerable arterial plaques that develop as a product of inflammatory processes initiated by high levels of LDL [1]. Thus, molecular imaging biomarkers of inflammation may play an important role in the monitoring the development and efficacy of new therapeutics as well as in the early detection of the disease. In this paper, we describe the development and evaluation of new cathepsin activatable agents and their comparison to the new *α*_*v*_ *β*_3_ integrin targeted agent in the detection of atherosclerosis associated inflammation in apoE^−/−^ mice using established FMT technology. We further demonstrate the sensitivity of individual biomarkers to detect inhibition of inflammation in prophylactic studies with the therapeutic ezetimibe.

We have developed new cathepsin agents, ProSense FAST and Cat B FAST, based on low molecular weight peptides flanked by fully quenched fluorophores. Once the protease cleaves the low molecular weight peptide, the fluorophores diffuse apart leading to a full fluorescence signal. These agents reach maximum activation within six to eight hours, while the polymer-based agent ProSense [27, 37] requires 24 hours for full activation. Activation of ProSense FAST is nonselective for tested cathepsins similar to the polymer-based ProSense, which is only weakly selective for cathepsin B. Conversely, the Cat B FAST agent is highly selective for cathepsin B. Thus, parallel measurements using the new agents and polymer-based ProSense can address the effect of the protease selectivity and to some extent also the potential effect of the polymer coil on the detection sensitivity of atherosclerosis associated inflammation. 

The *α*_*v*_ *β*_3_ integrin targeted agent, IntegriSense, has been used successfully in the detection of tumor growth [46] and RGD-based *α*_*v*_ *β*_3_ integrin targeted agents in the detection of inflammation and angiogenesis in mouse and rabbit models of atherosclerosis [40–42]. We evaluated the sensitivity and specificity of IntegriSense in the detection of atherosclerotic lesions in the apoE^−/−^ mice model of atherosclerosis and compared it to the cathepsin-activatable agents. All new agents were developed with the requirements for the design of low molecular weight therapeutics. Fluorophores were selected to align with the parameters needed to stay within the optimal tissue transparency window (680–950 nm) [27, 30], with potential for future successful applications in the clinical evaluation of patients. 

We employed FMT technology for the quantitative *in vivo* detection of molecular imaging biomarkers in both apoE^−/−^ and control C57BL/6 mice and confirmed the localization of the measured fluorescence using CT. We found that the FMT measured concentrations of imaging biomarkers in the thoracic area of apoE^−/−^ mice indicate that these agents accumulated in the aortic arch and the roots of adjacent carotid and subclavian arteries (sites of histologically confirmed atherosclerosis) similar to previous reports [37]. Moreover, the *in vivo* measured agent concentrations of both cathepsin-activated and *α*_*v*_ *β*_3_ integrin targeted agents correlated well with the *ex vivo* fluorescence in the same areas of the dissected arteries of apoE^−/−^ mice. 

An early stage of atherosclerotic lesions is characterized by infiltration of monocytes and macrophages and their development into foam cells [1–3] controlled by extracellular receptors including *α*_*v*_ *β*_3_ integrin [19]. As the atherosclerotic plaque progresses, endothelial cells, macrophages, and foam cells die, releasing matrix metalloproteinases and other intracellular enzymes such as cathepsins [20–22, 24]. The cell proliferation triggers on *α*_*v*_ *β*_3_ integrin-dependent angiogenesis, originating from adventitial vasa vasorum and resulting in immature, fragile, and leaky vasculature, triggering intraplaque hemorrhage [25, 26]. To understand the cellular profile of the imaging biomarkers, we conducted flow cytometric analysis of cells isolated from dissected aortic tissues of apoE^−/−^ mice and showed that cathepsin agents were activated primarily in myeloid (monocytes and macrophages) cells while the *α*_*v*_ *β*_3_ integrin agent was found not only in myeloid cells but also in lymphoid and stromal (endothelial) cells indicating that IntegriSense likely detects inflammatory cells, angiogenesis, and cell proliferation. Flow cytometry of blood from animals injected with cathepsin agents detected very low levels (compared to aortas) of the agents in both myeloid and lymphoid cells 24 hours after agent injection, while *α*_*v*_ *β*_3_ agent was also detected in both cell types, albeit at much higher levels for myeloid cells, indicating again involvement of *α*_*v*_ *β*_3_ integrin in the development and proliferation of inflammatory cells.

Immunofluorescence detected the activated ProSense agent in the center of the atherosclerotic lesions, some of it co-localized with resident macrophages confirming that cathepsins are activated in the inflammatory cells and later released from the dead cells into the core of the plaque. The *α*_*v*_ *β*_3_ agent was found in advanced atherosclerotic lesions densely distributed in the adventitia and some was also co-localized with macrophages within the core of the lesions reflecting expected development of new vasculature and already discussed expression on the inflammatory cells. FMT measured concentrations of cathepsin agents ProSense and Cat B FAST in apoE^−/−^ mice correlated well with the number of macrophages detected in the six different areas of the aortic arch, supporting the flow cytometric and immunohistochemistry data. However, the correlation between the macrophage count and FMT measured concentrations of *α*_*v*_ *β*_3_ agent was poor, which was expected giving the high levels of *α*_*v*_ *β*_3_ on the other cells types within the plaque. 

Co-localization of cathepsins (B, L, S and K) and macrophages in the core of atherosclerotic lesions of apoE^−/−^ mice and human patients has been documented many times [20–24, 28, 29, 31, 34, 35], confirming an important role of these proteases in the progression of atherosclerosis associated inflammation and the development of vulnerable plaques. Activation of newly developed cathepsin agents, ProSense FAST and Cat B FAST and previously studied [34, 35, 37] polymer-based, ProSense, in atherosclerotic lesions of apoE^−/−^ mice can therefore potentially serve as an imaging biomarker of inflammation. 

The co-localization of *α*_*v*_ *β*_3_ integrin agent with macrophages and its localization in the adventitia agree well with the previously documented role of *α*_*v*_ *β*_3_ integrin in the transformation of monocytes and macrophages in the core of atherosclerotic lesions [19, 39–42] and with the role of this integrin in the neoangiogenesis of vulnerable plaques [26, 42, 43]. The *α*_*v*_ *β*_3_ integrin agent is therefore, contrary to cathepsin agents, a molecular imaging biomarker of both inflammation and angiogenesis. The poor correlation between the FMT measured *α*_*v*_ *β*_3_ agent concentrations and the macrophage counts may indicate binding of this agent to cells other than inflammatory cells within the atherosclerotic lesions. Binding to myeloid cells within the blood may also influence the FMT signal.

Elevated levels of LDL in the blood of apoE^−/−^ mice on regular chow and even greater in mice on chow containing elevated levels of fat and/or cholesterol translate into the formation of atherosclerotic lesions in the aortic arch and proximal arteries as early as 3 months after the birth [44, 45, 47]. The onset of the disease and the size of the lesions varied between individual animals. FMT *in vivo* measurements of apoE^−/−^ mice fed with a high cholesterol diet found, when compared to C57BLK/6 mice fed with regular chow, elevated levels of the cathepsin probe activation as early as 15–20 weeks after diet initiation. The levels of activation started to grow exponentially after 25 weeks with an increasing variability among individual animals. In a similar set of FMT measurements, the accumulation of *α*_*v*_ *β*_3_ agent in atherosclerotic lesion of apoE^−/−^ mice was detected two to five weeks later and then the concentration of the agent was again increasing exponentially. Our data indicate a good correlation between the activation of cathepsin agents and atherosclerosis-associated inflammatory processes in a longitudinal study using these agents which may provide a noninvasive early means of detection of inflammation in developing atherosclerotic lesions of apoE^−/−^ mice. The somewhat delayed onset of the FMT measured *α*_*v*_ *β*_3_ agent accumulation might be the result of the later onset of plaque associated angiogenesis. Since both elevated inflammation as well as angiogenesis are characteristics of vulnerable plaques, both cathepsin as well as *α*_*v*_ *β*_3_ integrin agents may serve in their *in vivo* detection. 

Ezetimibe, a selective inhibitor of intestinal cholesterol absorption, has been shown to reduce plasma LDL, increase HDL, and inhibit atherosclerosis associated inflammation [44, 45, 48]. We found that all cathepsin agents at some point detect inhibition of inflammation by ezetimibe in prophylactic longitudinal study of apoE^−/−^ mice fed on high cholesterol diet. However, to our surprise, the new and nonselective fast acting agent ProSense FAST showed a statistically significant difference only at the 12 week time point, while the polymer-based ProSense detected the statistically different inhibition only at the 31-week time point. The most sensitive for the detection of inhibition by ezetimibe was Cat B FAST, finding the statistically significant inhibition at both 21 and 31 weeks. This may indicate that the selectivity rather than the size of the agent, as might be concluded from previous studies [37], is critical for an efficient detection of atherosclerosis-associated inflammation using cathepsin probes. FMT measurements with the *α*_*v*_ *β*_3_ integrin selective agent did not detect ezetimibe-induced inhibition of atherosclerosis progression in apoE^−/−^ mice. This is likely a reflection of a broader spectrum of processes involved in the accumulation of *α*_*v*_ *β*_3_ agent in the atherosclerotic lesions. Some of these processes may not be affected by the treatment with ezetimibe or they are affected on different time scales. Hence, a more detailed analysis of the functions of these two types of imaging biomarkers may contribute new details on the development of atherosclerotic lesions. 

## 5. Conclusions

Here we describe the functional *in vivo* imaging evaluation of new cathepsin activatable and *α*_*v*_ *β*_3_ integrin targeted agents, prepared using requirements for the design of new therapeutics. Using FMT, we measured quantitative changes in atherosclerosis-associated inflammation in apoE^−/−^ mice and compared these to the previously studied polylysine-based cathepsin agent. We have demonstrated the importance of cathepsin agent selectivity and capability of all agents to quantitatively detect longitudinal progress in inflammation and angiogenesis of atherosclerotic lesions. We have further showed that cathepsin agents detect inhibition of inflammation as a result of prophylactic treatment with the anti-inflammatory therapeutic, ezetimibe [44, 45].

## Figures and Tables

**Table 1:** Measurement of IntegriSense selectivity in binding to different integrin receptors IC_50_ values (mean ± SEM) of IntegriSense 750 and Parent Compound in cell adhesion assays.

	IC_50_ (nM)	
	IntegriSense 750	Parent compound
*α*_ v_ *β*_ 3_	3.5 ± 0.2	0.82 ± 0.2
*α*_ v_ *β*_ 5_	41 ± 12	1.3 ± 0.2
*α*_ IIb_ *β*_ 3_	48 ± 15	>10,000
*α*_5_ *β*_1_*	>5,000	>10,000

^∗^Binding assays to vitronectin were performed for all integrin receptors except *α*_5_ *β*_1_ which was performed on fibronectin.

**Table 2:** Serum cholesterol levels and plasma ezetimibe concentration (Mean ± SD) of ezetimibe treated and control apoE^−/−^ mice.

Group	HDL-C (mg/dL)	(LDL + VLDL)-C (mg/dL)	Ezetimibe (*μ*M)
Control	11.5 ± 0.9	411 ± 59	Undetectable
Treated	32.5 ± 2.9	106 ± 27	0.55 ± 0.11
